# Experimental evaluation of the cross-protection between Sheeppox and bovine Lumpy skin vaccines

**DOI:** 10.1038/s41598-020-65856-7

**Published:** 2020-06-01

**Authors:** Jihane Hamdi, Zahra Bamouh, Mohammed Jazouli, Zineb Boumart, Khalid Omari Tadlaoui, Ouafaa Fassi Fihri, Mehdi EL Harrak

**Affiliations:** 1 Research and Development Virology, Multi-Chemical Industry, Lot. 157, Z I, Sud-Ouest (ERAC) B.P.: 278, Mohammedia, 28810 Morocco; 2 Institut Agronomique et vétérinaire Hassan II, Rabat, Morocco

**Keywords:** Viral infection, Live attenuated vaccines

## Abstract

The Capripoxvirus genus includes three agents: Sheeppox virus, Goatpox virus and Lumpy skin disease virus. Related diseases are of economic importance and present a major constraint to animals and animal products trade in addition to mortality and morbidity. Attenuated vaccines against these diseases are available, but afforded cross-protection is controversial in each specie. In this study, groups of sheep, goats and cattle were vaccinated with Romania SPPV vaccine and challenged with corresponding virulent strains. Sheep and cattle were also vaccinated with Neethling LSDV vaccine and challenged with both virulent SPPV and LSDV strains. Animals were monitored by clinical observation, rectal temperature as well as serological response. The study showed that sheep and goats vaccinated with Romania SPPV vaccine were fully protected against challenge with virulent SPPV and GTPV strains, respectively. However, small ruminants vaccinated with LSDV Neethling vaccine showed only partial protection against challenge with virulent SPPV strain. Cattle showed also only partial protection when vaccinated with Romania SPPV and were fully protected with Neethling LSDV vaccine. This study showed that SPPV and GTPV vaccines are closely related with cross-protection, while LSDV protects only cattle against the corresponding disease, which suggests that vaccination against LSDV should be carried out with homologous strain.

## Introduction

Capripoxvirus genus includes 3 viruses, namely Sheeppox virus (SPPV), Goatpox virus (GTPV) and Lumpy Skin Disease Virus of cattle (LSDV). Diseases related to these viruses are of economic importance as they cause significant damages on meat, milk production, wool and leather quality in addition to carcass depreciation and mortality^1–3^.

SPPV and GTPV are widespread throughout Northern and Central Africa, Middle East, Indian subcontinent, Central Asia, China, Vietnam and Russia^4,5^. Recent outbreaks have been reported in Azerbaijan, Greece and Bulgaria^5^. LSDV was first confined in Austral Africa then spread to Middle East areas including Israel, Lebanon, Jordan, Kuwait and Saudi Arabia. Since 2013, the disease has been notified in Turkey, Iran, Cyprus, Greece, Azerbaijan, Georgia, Russia, Bulgaria, Serbia, Albania, Kazakhstan, China, Bangladesh and India^4,6,7^.

All capripoxviruses are antigenically related to each other and can only be distinguished by molecular characterization^8,9^. Though SPPV and GTPV are serologically indistinguishable, some authors have reported a host preference with most strains of SPPV and GTPV^10^.

Vaccination is the only effective method to control the disease in endemic areas. Despite vaccination efforts, only a few countries have successfully eradicated these diseases^11^. To date, available vaccines are live attenuated specific for small ruminants and cattle. In some countries, people use SPPV to vaccinate against GTPV and LSDV^12^. Cross-protection between SPPV and GTPV has been already demonstrated for various strains and vaccination with LSDV Kenyan Sheep and Goat pox (KSGP) strain has also been widely used for many years to protect small ruminants against SPPV and GTPV^13,14^.

Experiments have been performed in some countries to evaluate the cross-protection amongst the 3 members of Capripoxvirus genus^15^. Immunological studies and field trials have been conducted, but few studies assessed the efficacy of vaccine strains by challenge^16,17^.

The aim of this study was to evaluate the cross-protection between SPPV, GTPV and LSDV on sheep, goats and cattle. We selected a well-known, highly immunogenic SPPV strain (Romania) and the most used LSDV strain (Neethling) to vaccinate sheep, cattle and goats against their respective diseases. Protection was evaluated by serological monitoring, using neutralizing antibody assays and challenge using corresponding virulent strains.

## Results

### Vaccine preparation

For the live Romania SPPV vaccine, the virus showed a cytopathic effect starting at day 3 (D3) and was harvested at D4 post-infection (pi) with a titer of 10^6.0^ TCID_50_/ml. After freeze-drying, the infectious titer of the final vaccine was 10^5.0^ TCID_50_/vial of 100 doses.

The Neethling vaccine strain showed similar effect starting from D3 and was harvested at D5 pi. The infectious titer was 10^6.8^ TCID_50_/ml at the harvest and 10^5.7^ TCID_50_/ vial of 50 doses after vaccine freeze-drying.

Vaccines complied with analytical QC testing. They were sterile, free from adventitious agents and no extraneous agents were detected by qPCR.

### Animal vaccination

In the group of sheep vaccinated with Romania SPPV (G1), a slight rise in temperature was noticed 5 to 7 days post vaccination (pv) in 2 sheep with limited inflammation at the injection site. The other sheep remained within the normal range. In the group of goats vaccinated with Romania SPPV (G2), 2 weeks following vaccination, the body temperature remained in the normal range and no clinical signs of GTPV nor inflammation at inoculation site were observed. Cattle vaccinated with Romania SPPV (G3) did not show hyperthermia nor inflammation at the injection site.

Sheep vaccinated with LSDV vaccine (G4) showed a slight increase in temperature between D2 and D4 pv and a limited local reaction was observed in 5 out of 8 animals from D3 pv until D12 pv. Cattle vaccinated with LSDV Neethling (G5) did not show hyperthermia except a slight rise at D7 pv for one cow.

Serology of vaccinated animals, evaluated by Virus Neutralization Test (VNT) revealed 100% of positive animals starting from D14 pv in the group of sheep vaccinated with SPPV Romania (G1), with a maximum titer of 1.6 log_10_ at D21 pv. In the group of goats (G2), 5 out of 8 goats were positive at D21 pv with a maximum titer of 1.7 log_10_. Serology of cattle vaccinated with SPPV Romania SPPV (G3) showed no seroconversion as all the animals remained negative.

**Figure 1:**
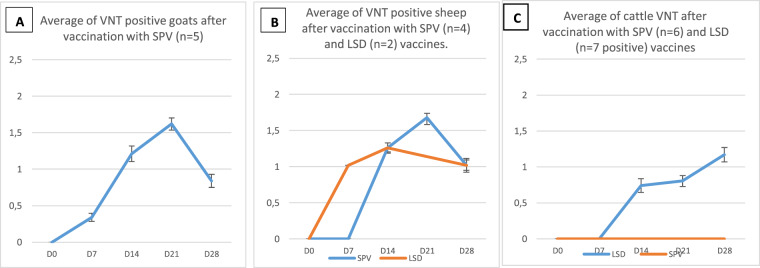
Serological response of animals after vaccination. Graph A presents average antibody titers of positive goats vaccinated with SPV vaccine. Graph B presents average antibody titers of positive sheep vaccinated with LSD and SPV. Graph C presents average antibody titers of cattle vaccinated with SPV and LSD vaccines.

In the group of sheep vaccinated with LSDV vaccine (G4), only 2 among 8 animals were positive using VNT, starting from D7 pv with a low neutralizing antibody titer (0.3 log_10_). In the group of cattle vaccinated with LSDV vaccine (G5), 50% of animals were seropositive at D28 pv (Fig. 1).

Control animals remained negative during the observation period.

### Challenge virus preparation

Sheep used to prepare the SPPV challenge strain showed a huge skin inflammation of 10 cm in diameter at the injection site. Animals were euthanized at D13 pi to collect aseptically lymph and inflammatory tissues. Viral suspension showed a CT value of 13,3 at qPCR, which corresponds approximately to a titer of 10^7.0^ TCID_50_/ml.

**Figure 2:**
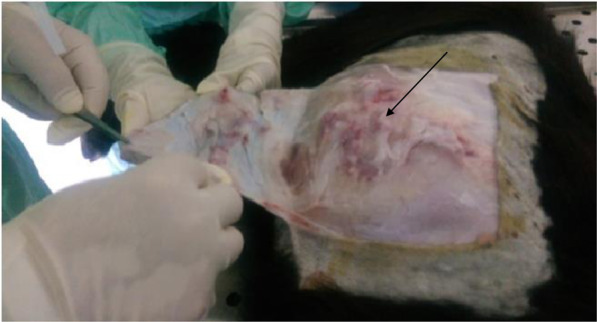
Inflammatory tissue after inoculation of the goat with the virulent goatpox strain and necropsy. The arrow shows the inflammatory tissue collected.

Under the same conditions, goats inoculated with virulent GTPV showed an important erythema followed by a skin inflammation that turned into oozing pustule (Fig. 2). Animals were euthanized at D12 pi and the collected tissue was prepared following the same protocol as SPPV. The CT of the supernatant, obtained by qPCR, was 18,9 which corresponds to 10^5.5^ TCID_50_/ml

The titer of LSDV challenge virus used in Sciensano was 10^6.55^ TCID_50_/100 μl.

### Experimental infection

After the challenge of sheep with virulent SPPV strain, unvaccinated sheep showed an increase in body temperature between D4 and D10 pi reaching 41.7 °C at D5 pv, in addition to a local reaction at the injection site that appeared at D3 pi. Typical SPPV skin secondary nodules were observed at D10 pi.

Sheep vaccinated with Romania SPPV (G1) exhibited a transient increase in temperature between 39.6 °C and 39.7 °C at D6 and D7 pi. Hypersensitivity reaction at the injection site in the whole group was noticed at D2 pi in the first dilution and disappeared the following day. None of the immunized animals showed clinical signs of SPPV during the observation period. Serological response confirmed the identity of the challenge virus as demonstrated by the increase of neutralizing titer at D14 pi (2.2 log_10_). Using the protection index protocol, the infectious titer obtained in unvaccinated sheep was 10^5.7^ ID_50_/ml, significantly higher than that obtained with vaccinated animals (10^0.75^ ID_50_/ml) (*P*  ≤  0.05). The protection index (PI) was estimated at 4.7 in sheep vaccinated with Romania strain (G1).

Sheep vaccinated with LSDV vaccine (G4) showed an increase in body temperature between D7 and D8 pi. Hypersensitivity reaction at the injection site was observed at D2 and D3 pi (n = 7), followed at D4 pi, by papules that reached 3 cm of diameter in 3 among 8 vaccinated animals. Two among 8 vaccinated sheep showed a PI of 3 and 4.5, while 3 sheep showed moderate protection (PI: 1, 1 and 2). The other 3 animals did not show any protection.

In the goat group, the 2 unvaccinated control goats challenged with the virulent GTPV virus showed a rise in mean body temperature, between D4 and D9 pi, that reached 41.1 °C at D6 pv. Typical skin papules appeared at injection site at D3 pi and reached 2 cm of diameter at D5 pi. Lesions showed confluence tendency and evolved to eschar and abscess in low dilution range. Secondary lesions were also observed in hairless part of the body at D10 pi.

**Figure 3:**
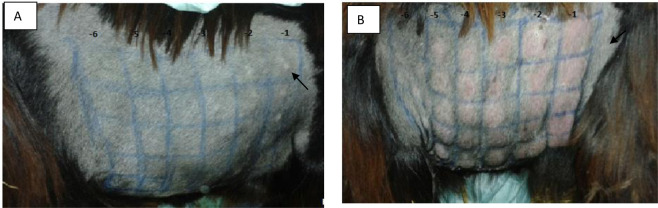
(**A**) Figure of challenged vaccinated goat showing hypersensitivity reaction and no local inflammations on site of inoculation with10–1 to 10–6 dilutions (left to right) of virulent GPV. (**B**) figure of challenged unvaccinated goat showing local inflammation on site of inoculation. The arrows show inflammation or hypersensitivity reaction on inoculation site.

Goats vaccinated with Romania strain (G2) did not show any rise in body temperature, however, a hypersensitivity reaction at the injection site was observed during 2 days pi (Fig. 3). The reaction disappeared completely before D4 pi. None of the immunized animals showed clinical signs of GTPV during the observation period. Serology confirmed the identity of the challenge virus since all the animals showed an increase of the neutralization titer (2.9 log_10_) at D7 pi. The obtained titer in the 2 unvaccinated animals was 10^6.5^ID_50_/0.2 ml, significantly higher than that obtained with vaccinated animals (10^1.3^ID _50_/ml) (*P*  ≤  0.05). The PI was 5,2.

All cattle vaccinated with SPPV Romania vaccine (G3) showed, after challenge with virulent LSDV virus, fever with spikes between D7 and D9 pi. Three out of 7 cattle showed important swelling starting from D9 pi and developed localized nodules in one animal and generalized in 2 animals, which developed large pre-scapular lymph node enlargement. The 3 animals were viremic after challenge (Ct = 32). Animals of the control group displayed fever until the end of the trial with a spike at D6 to D8 pi. Three out of 5 animals were viremic starting from D3 pi and developed generalized nodules starting from D13 pi. Regarding the clinical score, there was no significant difference between groups of unvaccinated and vaccinated cattle with Romania SPPV (*P* > 005).

In the group of cattle vaccinated with Neethling strain (G5), body temperature remained normal and no animal showed clinical signs nor viremia after challenge. Unvaccinated control cattle showed pathognomonic clinical signs of LSDV in 3 out of 5 animals with viremia (between 3 and 17 dpi) and positive buccal swabs. Clinical scores were significantly higher in the group of unvaccinated animals compared to cattle vaccinated with Neethling strain (*P*  ≤  0.05) (Table 1).

**Table 1:** Seroconversion and Protection on sheep, goats and cattle, confered by vaccination with SPPV Romania and LSDV Neethling.

	Sheep		Goats		Cattle	
	Seroconversion	Protection	Seroconversion	Protection	Seroconversion	Protection
SPPV (Romania)	4/4	4/4	5/6	4/4	0/6	4/7
LSDV (Neethling)	2/8	2/8 + +3/8+	NT	NT	7/15	7/7

## Discussion

Prevention of Capripoxvirus infections in countries where the diseases exist is mainly based on vaccination of susceptible animals^18^. As the 3 capripoxviruses share major neutralization sites, cross-immunity has been reported, but not experimentally documented^4,19^.

The use of SPPV vaccine to protect sheep, goats and cattle against infections is the most common^4,20^. SPPV vaccine strains, such as the Romania SPPV and Yugoslavian RM65, are widely used in endemic countries to protect sheep against their disease,^13,21–23^. Romania SP strain has also been used to vaccinate goats with controversial results. Rao *et al*. (2000) and Abdelfatah *et al*. (2018) reported the vaccine to be not protective of goats against GTPV^24,25^. In Saudi Arabia, Abuelzein *et al*. (2003) noticed the apparition of Goatpox disease in animals vaccinated with locally produced Romania SPPV vaccine^26^, whereas the same authors recommended vaccination of goats with the same vaccine at 3 months of age with annual booster^27^. According to Molla *et al*. (2017), immunization failure may be linked to vaccination conditions, vaccine reconstitution and coverage rate^28^. However, although no challenge was carried out to confirm the cross protection, Abdelfatah *et al*. (2019) observed that vaccination of goats with Romania strain induced cell-mediated immunity with a satisfactory PBMC and lymphocyte proliferation level^29^.

To vaccinate cattle against LSDV, partial cross-protection with SPPV vaccines has been documented from the field in the Middle East and Africa with SPPV RM65, Bakirkoy and Romania strains^4^. Vaccination failure after the use RM65 strain in cattle has been cited by Brenner *et al*. (2009) and Abutarbush *et al*. (2014). Indeed, during the LSDV outbreak of 2006 in Israel, an incomplete protection of herds was detected, as 11% of vaccinated cattle showed LSDV symptoms^12^. The same results were reported in Jordan (Abutarbush *et al*., 2014) where a partial protection was provided by RM65 vaccine, although morbidity and mortality rates were lower compared to unvaccinated herds^30^. In another study, it appeared that 10 time doses of RM65 vaccine induced high antibody response, but no challenge has been performed to evaluate cell-mediated protection^17^. Sevik and Dorgan (2016) also reported failure of vaccination with SPPV Bakirkoy strain to protect cattle in the field against LSDV^31^. Similarly, Abdallah *et al*. (2018) and Zeedan *et al*. (2019) described cases of infected cattle emerging from a herd vaccinated with Romania vaccine in Egypt^32,33^. Studies on humoral and cellular response upon vaccinated cattle against LSDV with Romania strain showed an induction of humoral response, proliferation of lymphocytes and stimulation of Interferon gamma and IL4, but no challenge was carried out to confirm the protection^15,34,35^. Also, Mikhael *et al*. (2016) did not perform any challenge, however they observed that Romania SPPV vaccine did not protect sufficiently cattle against LSDV and concluded on the need to use homologous strain for cattle vaccination^36^. In another study, Mikhael *et al*. (2017) reported that combination of SPPV and GTPV may induce better protection of cattle against LSDV but the homologous strain remains the best^37^. Similar results were reported by Aboul Soud *et al*. (2018), who showed that no serological response was induced in cattle vaccinated with Romania strain while a trivalent Capripox vaccine (made up of SP Romania, GTPV Held and KSGP 0180) induced antibodies in 66% of vaccinated animals^38^.

Vaccination of sheep with GTPV vaccine has been rarely reported. Agrawal *et al*. (1997) described a weak protection at the challenge on a group of 6 sheep vaccinated with a not named strain of GTPV^39^. Nevertheless, protection of cattle vaccinated with Kedong and Isiolo GTPV has been reported against LSDV challenge^40^. The GTPV Gorgan strain is also commonly used in Iran to protect cattle against LSDV along with SPPV RM65^35^ and in a recent experiment, Gorgan strain has been reported to be more immunogenic than KSGP 0180 and Neethling strains of LSDV^41^. This experimental study was based on challenge and cellular immune response (by Delayed-Type Hypersensitivity). Such observations should however be confirmed, as protection may depend on the replicative capacity of the virus in the animal and on the vaccine production process.

The use of LSDV to prevent infections of sheep and goats has not been reported to our knowledge. However, Kenya LSDV strain (long time considered as a Sheep Goatpox (SGP) has been extensively used to protect small ruminants against their respective diseases with controversial results. The KSGP 0180 and KSGP 0240 have been reported to induce incomplete protection of cattle against LSDV in Egypt and Ethiopia^42–44^. In sheep, similar vaccination failure has also been reported against SGPV using the KSGP O-180 strain^4^.

The discrepancy reported on cross-protection between *Capripoxvirus* genus members prompted us to conduct this experiment on a vaccination/challenge study using SPPV vaccine (Romania strain) to protect sheep, goats and cattle against SPPV, GTPV and LSDV respectively. In parallel, we also conducted a LSDV (Neethling strain) vaccination trial to protect sheep and cattle against SPPV and LSDV. Strains used in this experiment, have been tested before to be immunogenic in target species at the recommended dose of vaccination.

We chose live vaccines as they are the most common in the field and known to confer a solid immunity when used properly for the target species^17,45^.

Romania SPPV strain was selected because it has been used worldwide to prevent infections in small ruminants and is known to confer a high level of protection in sheep^46^. When used for mass vaccination, results are conclusive in the field and if vaccination pressure is regularly maintained, it may lead to disease eradication in the country^47,48^. Romania SPPV vaccine strain grows very well on different primary cells and also on Vero cell line which are suitable for vaccine preparation to avoid unavailability and potential adventitious contaminants. However, uncontrolled serial passages of virus on continuous heterologous cell line like Vero cells may limit the capacity of the strain to replicate in animals, affecting its immunogenicity^49^. This phenomenon has been observed with KSGP and Neethling strains, passed several times on Vero cells, that showed to be ineffective to protect cattle against the infection^50^. Regarding LSDV, Neethling strain is widely used and has been involved in the eradication of the disease in many countries, despite post-vaccination reported effects (Neethling disease)^51^.

To vaccinate animals against Capripoxvirus diseases, the minimal recommended vaccine dose is 10^2.5^ TCID_50_ for small ruminants and 10^3.5^ TCID_50_ for cattle^23,52,53^. In our study, we used a dose of 10^3.0^ TCID_50_ for sheep and goats and 10^4.0^ TCID_50_ for cattle, which are the most common used doses, to secure replication of the vaccine strain in animals. Used vials for animal vaccination were titrated on cells to ensure that the animals received the right dose.

Vaccination monitoring was conducted using VNT that detects protective IgG specifically. Sheep vaccinated with Romania SPPV strain were all seropositive at D14 pv with a maximum neutralizing titer of 1.6 log_10_ and 5 out of 8 goats vaccinated with Romania strain were positive at D21 with a maximum neutralizing titer of 1.7 log_10_. The reported kinetics of antibody response showed an increase in the titer despite the fact that Capripoxviruses induce mainly a cell-mediated immunity^15,35^. Those results are in agreement with Bhanuprakash *et al*. and Boumart *et al*., who cited an increase in neutralizing antibodies between D14 and D21 pv^54,55^.

The challenge of small ruminants vaccinated with SPPV Romania and LSDV Neethling was conducted according to the protocol by Fassi-Fehri *et al*.^56^. This method allows quantitative assessment of the conferred immunity and is based on the titers obtained from the challenge virus in vaccinated and unvaccinated animals. We selected this method because it is the one used routinely for years to conduct potency testing for SPPV vaccines. The method works perfectly for sheep and goats.

During the observation period, unvaccinated sheep and goats showed typical symptoms of SPPV and GTPV respectively. Unvaccinated animals were euthanized at D12 pi because of symptom severity, and virus recovery was conducted successfully from skin lesions in both sheep and goats. Serology also showed increase in the VNT titer after challenge, confirming the use of the homologous virulent strain in each species. The challenge dose allowed virus titration in sheep and goats and comparison between high and low immunogenic vaccines. Protection index in vaccinated sheep and goats was between 4.7 and 5.2, giving evidence of complete and long-lasting protection in those species against SPPV and GTPV infections^56^.

In the group of sheep vaccinated with LSDV Neethling, only 2 animals among 8 were serologically positive with a low antibody titer. The challenge showed partial protection, as only 2 animals were fully protected (positive also in serology), 3 partially protected and 3 others not protected. To our knowledge, this is the first time an experiment was carried out to test efficacy of Neethling strain in sheep. Challenge of goats vaccinated with LSDV was not performed in this experiment.

In cattle, vaccination monitoring using VNT showed 50% of positive cattle vaccinated with LSDV while no antibody response was detected in cattle blood three weeks after vaccination with SPPV vaccine. The discrepancy between cattle and small ruminants has been reported by many authors, who highlighted the fact that cattle do not all (100%) react to vaccination^41,57,58^, suggesting that a challenge is required to confirm protection of cattle against the disease. To perform challenge on cattle against LSDV virulent strain, Sciensano laboratory adopted a semi-quantitative method. Challenge of LSDV Neethling vaccinated cattle revealed full protection as no animal showed symptoms of LSDV, while 3 out of 5 control animals displayed numerous nodules accompanied by fever. Partial protection was observed in vaccinated animals with Romania strain as 4 out of 7 challenged calves showed protection while 3 animals displayed fever, viremia and typical lesions of LSDV. Our study demonstrated partial protection against LSDV when we used SPPV vaccine, which complies with Michael *et al*. observations^59^. The control group showed infection of 3 among 5 challenged cattle, which is in agreement with the experiment by Gari *et al*., who described clinical disease in only 3 out of 5 animal controls^41^. Besides, previous authors reported that only half of the infected animals may show clinical signs of LSDV^60,61^.

Molecular analysis of the 3 Capripoxviruses was investigated by several authors. Tulman *et al*. (2002) sequenced and analyzed complete genomes of several strains of SPPV, GTPV and LSDV and reported presence of a parent LSDV-like virus^8^. These results were confirmed by Biswas *et al*. (2019) who analyzed 36 different strains and showed a loss of 5 ORFs in the SPPV/GTPV lineage^9^. In another study, analysis of partial fragment of B22R gene showed a specific deletion in SPPV Romania comparatively to GTPV and LSDV^62^. Rouby *et al*. (2018) also detected 21-nucleotide deletion within RPO30 gene of SPPV in general^63^. Many studies have confirmed that GTPV is closely related to LSDV than SPPV is to LSDV^64,65^, which can explain the protection conferred to cattle vaccinated by GTPV Gorgan strain against LSDV. Additional studies are required to support our conclusions.

Capripox infections are now considered as emerging diseases which threaten new geographic areas (austral Africa and Europe). In most of the endemic countries, there are mixed flocks with goats and sheep living in promiscuity and, until now, there is no universal solution to avoid capripoxvirus diseases spreading except vaccination. The current study showed that goats and sheep vaccinated with Romania SP vaccine are well protected against a challenge with virulent GTPV and SPPV strains. However, Neethling strain did not protect sheep against the disease after challenge. Cattle vaccinated with Neethling strain showed full protection against LSDV virulent strain, while vaccination with SPPV strain did not give full protection against infection with the virulent LSDV virus. Preferably, vaccination should be conducted with the homologous Neethling strain.

## Methods

### Vaccines preparation

The SPPV vaccine used for the experiment was prepared in MCI laboratory. It is a commercial vaccine based on Romania strain of ovine origin, attenuated by Precausta *et al*.^46^ and manufactured by MCI, Morocco. The vaccine strain has been shown to provide strong protection of sheep beyond 24 months^66^. The strain was sequenced in Sciensano and the Full Genome Sequence (FGS) confirmed the identity of the virus (Unpublished data). For the antigen preparation, the master seed virus was passed three times on primary testis cells maintained in Dulbecco’s Modified Eagle’s Medium (DMEM) with 10% irradiated fetal calf serum. The inoculation was carried out using a Multiplicity of Infection (M.O.I) of 0.01. Viral suspension was harvested when 80% of cytopathic effect (CPE) was observed, generally at day 4 pi. The viral suspension was then stored at −80 °C before use. Sterility, identity, purity testing and titration were carried out as part of the quality control of the intermediate product. The live vaccine was prepared from the virus suspension by addition of the stabilizer (4% peptone, 8% sucrose and 2% glutamate) and freeze-dried in LSI lyodryer. The vaccine was tested for sterility, identity, purity and infectious titer. To vaccinate, we used an animal dose of 10^3.0^TCID_50_ for small ruminants and 10^4.0^ TCID_50_ for cattle.

The LSDV Neethling attenuated strain of South Africa origin was used to prepare the live attenuated LSDV vaccine in our laboratory. This strain has been attenuated through 61 passages on chorio-allantoic membrane and has been used as a vaccine strain for decades in Africa, Middle East and Europe recently^52,67^. The identity of the Neethling vaccine strain we used was confirmed by full genome sequencing (unpublished data). For the antigen preparation, the master seed virus was passed three times on primary testis maintained in Dulbecco’s Modified Eagle’s Medium (DMEM) with 10% irradiated fetal calf serum. The viral suspension was harvested after 5 days of incubation at 35 °C. Sterility, identity, purity and titration were performed as part of the quality control of the intermediate product. The vaccine was freeze-dried according the same protocol applied for SPPV. To vaccinate, we used an animal dose of 10^3.0^ TCID_50_ for small ruminants and 10^4.0^ TCID_50_ for cattle.

For SPPV and LSDV vaccine reconstitution, a sterile diluent based on phosphate saline solution was used. Vaccines were injected directly after reconstitution.

### Vaccination with SPPV and LSDV vaccines

The experiment was conducted on animals housed in the high containment ABSL3. Animals were from Morocco local breeds, aged 6 to 8 months and tested negative by virus neutralization test (VNT) as described below.

Three groups of 4 sheep (G1), 8 goats (G2) and 13 cattle (G3) were subcutaneously (SC) vaccinated using Romania SPPV vaccine. Two groups of 8 sheep (G4) and 22 cattle (G5) were SC vaccinated using Neethling LSDV vaccine. All animals were monitored during 28 days for rectal temperature, clinical signs and inflammation at the injection site. Serum samples were collected weekly until D28 pv.

**Table 2:** Vaccination and method of challenge on animals vaccinated with SPPV Romania and LSDV Neethling vaccines.

Vaccination	Groups	Animals	Number	Dose	Challenge	Method
SPPV Romania	1	Sheep	4 V + 2 C	3	Virulent SPPV HELD	Protection index
	2	Goats	4 V + 2 C	3	Virulent GTPV VIETNAM	Protection index
	3	Cattle	7 V + 5 C	4	Virulent LSDV ISRAEL	Clinical scoring
LSDV Neethling	4	Sheep	8 V + 2 C	3	Virulent SPPV HELD	Protection index
	5	Cattle	7 V + 5 C	4	Virulent LSDV ISRAEL	Clinical scoring

V: vaccinated animals.

C: unvaccinated control animals.

### Challenge virus preparation

Infection was carried out according to international guidelines described for the care and handling of experimental animals, chapter 7.8 of the Terrestrial Animal Health Code and Directive 2010/63/UE of the European commission^68,69^. The protocol was submitted and approved by the Internal Laboratory Committee.

To prepare the challenge SPPV virus, we used the virulent SPPV Held strain, a Turkish strain, passaged on lamb testis cells^56^ and provided by IAV Institute in 2014. The virus was prepared by SC inoculation of 10 ml of viral suspension into 2 sheep flanks to obtain a giant pustule. After 13 days, sheep were euthanized to collect the subcutaneous inflammatory tissue at the injection site. The collected tissue was minced using sterile scissors and grounded after adding 2 volumes of PBS with 5% of antibiotics (penicillin and streptomycin). The suspension was centrifuged at 3500 RPM for 30 minutes. The supernatant was then aliquoted and stored at −80 °C for further use.

To prepare the challenge GTPV virus, we used the virulent GTPV Vietnam strain, originated from an infected goat lung^10^ and obtained from IAH Pirbright UK in 2012. Preparation of the challenge virus was the same as described above.

The virulent LSDV Israeli field isolate (10^6.5^ TCID_50_/100 μl) was used to inoculate vaccinated and unvaccinated control cattle in the confined facilities of Sciensano laboratory in Belgium.

### Challenge test

Vaccine potency testing was carried out by a challenge trial, under ABSL3 conditions using the virulent SPPV and GTPV strains for small ruminants. Vaccine potency testing on cattle was subcontracted to the European Reference Laboratory, Sciensano, in Belgium. Animal experiments were carried out in accordance with the international guidelines for care and handling of experimental animals.

Four sheep of G1 and 8 sheep of G4 were challenged at D28 pv with a virulent SPPV strain.  Four goats from G2 were challenged at D28 pv with a virulent GTPV strain. Seven cattle from G3 and 7 cattle from G5 were challenged with a virulent LSDV strain. Two sheep, 2 goats and 5 cattle were kept unvaccinated and challenged by their respective virulent strains for challenge validation (Table 2).

Small ruminants were challenged using the protection index protocol^56^ which consists on a virus titration by intradermal injection of serial dilutions (10^−1^ to 10^−6^) on shaved flank, 5 inoculation points per dilution and 0.2 ml per injection in each animal. The obtained titer on vaccinated animals with average of the group is compared with the average titer obtained in unvaccinated animals. The protective index (PI) represents the difference between the 2 titers (in log). During the challenge trial, animals were daily observed for specific symptoms, temperature and local inflammation at the injection site. Serum samples were collected weekly for serology until D14 pi. Animals were all euthanized 2 weeks after infection (D14 pi); the control animals were euthanized when severe generalized symptoms appeared.

Cattle were challenged with virulent LSDV strain 21 days pv by intradermal (ID) (4 points of 250 µl each) and IV (2 ml) routes. Following inoculation, cattle were monitored for appearance of clinical signs during 21 days then euthanized. Clinical response in challenged animals was measured using clinical reaction score (CRS).

### Laboratory testing

Vaccinated animals were monitored weekly for antibody response by VNT as described in the OIE Terrestrial Manual^23,53^. Blood was collected before and weekly after vaccination into dry tubes, which were centrifuged. Serum was then harvested and stored until use. Obtained sera were heat inactivated and serial 1:3 dilutions were mixed with a constant dose of SPPV or LSDV virus then incubated for one hour. Cell suspension was then added (OA3: ATCC^®^ CRL-6546 or MBDK: ATCC^®^ CCL22) and CPE was observed after 7 days incubation. Positive control without serum and negative without virus as well as cell control are introduced in the assay for validation. The neutralizing antibody titer was calculated according to Reed and Muench method^70^.

Organs of euthanized animals were analyzed using qPCR. DNA was extracted from the inflammatory tissue, using ISOLATE II Genomic DNA Kit (Bioline) following the manufacturer’s instructions. The extracted DNA was amplified using TaqMan Universal PCR Master Mix. The reactions were run on the Applied Biosystems 7500. The test was performed in 96-well Optical Reaction Plates using primers described by Bowden *et al*.^71^. Amplification conditions were: 95 °C during 10 min; and 45 cycles of 95° 15 s and 60 °C 1 min. Data were analyzed using the 7500 System software. Results were generated by determination of the threshold cycle (CT).

### Statistical analysis

For statistical analysis, we started with the test of equality of variances (F-test) to check if the two treatments have the same variance, in order to choose the appropriate test for the average comparison. Where variances are equal, the test of equality of expectations would be used i.e. for two observations of equal variances. If not, the equality of expectations test for two observations of different variances would be used.

Differences between antibody titers obtained in vaccinated animals were tested for significance. Infectious and protective titers obtained post challenge in SPPV and GTPV vaccinated small ruminants and SPPV and LSDV vaccinated sheep were also tested for significance. Comparison of vaccinated and unvaccinated cattle was performed following clinical scoring obtained after challenge. Values of *P* ≤ 0.05 were considered significant.

### Ethics and consent to participate

Animal experiments were carried out in accordance with the international guidelines for care and handling of experimental animals described in chapter 7.8 of the Terrestrial Animal Health Code and Directive 2010/63/UE of the European commission. The protocol was submitted and approved by the Internal Ethic Committee for animal experiment in MCI santé animale.
